# Irrigation water productivity is more influenced by agronomic practice factors than by climatic factors in Hexi Corridor, Northwest China

**DOI:** 10.1038/srep37971

**Published:** 2016-12-01

**Authors:** Xiaolin Li, Xiaotao Zhang, Jun Niu, Ling Tong, Shaozhong Kang, Taisheng Du, Sien Li, Risheng Ding

**Affiliations:** 1 Center for Agricultural Water Research in China, China Agricultural University, Beijing, 100083, China

## Abstract

Quantifying the influence of driving factors on irrigation water productivity (IWP) is vital for efficient agricultural water use. This study analyzed contributions of agronomic practice and climatic factors to the changes of IWP, based on the data from 1981 to 2012 in Hexi Corridor, Northwest China. Cobb-Douglas production functions were developed by the partial least squares method and contribution rates of the driving factors were calculated. Results showed that IWP and its driving factors increased during the study period, with different changing patterns. IWP was significantly correlated with the agronomic practice factors, daily mean temperature and solar radiation of the crop growing period. The agronomic practice factors including irrigation, fertilization, agricultural film, and agricultural pesticide contributed 20.6%, 32.8%, 42.3% and 11.1% respectively to the increase of IWP; and the contribution rates of the climatic factors, i.e. daily mean temperature and solar radiation, are −0.9% and 0.9%. And the contributions of these factors changed in different sub-periods. It is concluded that agronomic practice factors influenced IWP much more than climatic factors. The improvement of IWP should rely on advanced water-saving technology and application of optimum (need-based) fertilizer, agricultural film and pesticide, ensuring efficient use of agronomic inputs in the study area.

With the growing problems of water resources, competition among water-consuming sectors is particularly becoming intense. Agricultural production is seriously affected by water shortage since agriculture is the largest water consumer^1^. Food security issues become more and more severe under changing climate, increasing population and decreasing water available for agricultural production^2^,^3^,^4^. Irrigated agriculture, the major contributor of agricultural production, faces the challenge of improving irrigation water use efficiency and meanwhile ensuring food security^4^,^5^. Under the premise of limited arable land, improving agricultural water use efficiency is the key measure to alleviate the contradiction between the increasing demand of agricultural production and the shortage of available water^6^,^7^.

In the arid region, irrigation is the dominant factor influencing agricultural production^8^. Irrigation water productivity (IWP), defined as the yield produced per unit of irrigation water use^6^, has become an important criteria which takes into account of both agricultural production and water use efficiency. It is a comprehensive indicator for revealing the management level of both irrigation and crop^9^,^10^,^11^. Increasing the value of IWP would not only alleviate the pressure of limited water resources but also ensure the food security^12^,^13^. Analyzing the impacts of driving factors on IWP helps to explore ways of improving IWP, which is significant for efficient water use and agricultural sustainable development.

IWP is affected by many factors^5^,^12^,^13^,^14^, which should be comprehensively balanced and considered. Identification of the main limiting factors and understanding correlations between IWP and its driving factors are helpful for improving IWP. Since IWP reflects the relationship between yield and irrigation water, factors influencing yield or irrigation certainly have impact on IWP. Zwart and Bastiaanssen^5^ held that variability of IWP was induced by differences in climate conditions, crops, soil properties and agronomic practices that were related to the soil-plant-water continuum. Climatic factors are important determinants to irrigation amount and crop production^15^,^16^. Temperature, solar radiation and precipitation have effects on crop growth-development and photosynthetic capacity^16^,^17^,^18^. Species and varieties of crops will greatly determine water use and crop yield. Among different species of crops, water and nutrient use efficiencies are quite different; for instance, C_4_ plants have higher water productivity than C_3_ plants. And for the same species, water use efficiencies are also different among different varieties^12^. Soil properties, e.g. soil texture and organic matter content, play significant roles on soil water status and crop growth and thus affect crop yield and water productivity^12^,^19^. Agronomic practices include crop management, irrigation management and soil management, and so on^5^,^6^,^20^,^21^,^22^,^23^,^24^. Crop management contributes for obtaining high water productivity, such as appropriate cultivation, selection of varieties, weeding, etc. Technique, amount and timing of irrigation certainly influence IWP. For instance, deficit irrigation has been proposed as a measure of improving the IWP^20^. Hatfield *et al*.^19^ reviewed the effects of soil management on water productivity, including mulching, soil nutrient improvement, etc. Mulching, as one of soil surface modifications, changes the soil water status and is found to have positive effect on water productivity. Soil nutrients directly influence the crop photosynthesis^19^, and proper soil nutrients will improve water productivity. Hu *et al*.^25^ ranked the driving factors of IWP for the oasis wheat by using grey relational analysis in the middle reaches of Heihe River, Northwest China. It was found that the pesticide, chemical fertilizer and accumulated temperature were the main influencing factors of IWP.

Most of the studies focused on assessing the influence of a certain factor by controlling other variables or ranking the influence degrees of different driving factors at the region scale. Considering the combined and uncertain effects of the driving factors, and the spatial variability of the driving factors and influence degrees, it is necessary to analyze and quantify the influences of the driving factors that may affect IWP for improving IWP in the study area. Many researches have been conducted on the theories and methods for determining contribution rates. In addition to common statistical methods, e.g. regression analysis, production function method is widely used. Cobb-Douglas (CD) production function is often used to calculate the contribution rates of technology progress and economic growth factors, for it is convenient to use, easy to understand and accurate in estimation^26^,^27^,^28^. So far, quantitative estimation of the contribution rates of the driving factors on the changes of IWP has been rarely reported.

The Hexi Corridor is an important agricultural production base in the arid region of Northwest China, which highly relies on irrigation. Thus, in this region, analyzing the IWP and quantifying the influence of its driving factors can provide insights for efficient water use. The objectives of this study are to: (1) analyze the temporal changes of IWP for cereal crops and its driving factors over the past 32 years (1981–2012); (2) explore the correlations between IWP and its driving factors including the agronomic practice factors and climatic factors; and (3) establish PLS-CD production function models in different periods to quantitatively assess the contribution rates of the agronomic practice factors and climatic factors to IWP, as to provide scientific basis for improving regional IWP.

## Materials and Methods

### Study area

**Figure 1:**
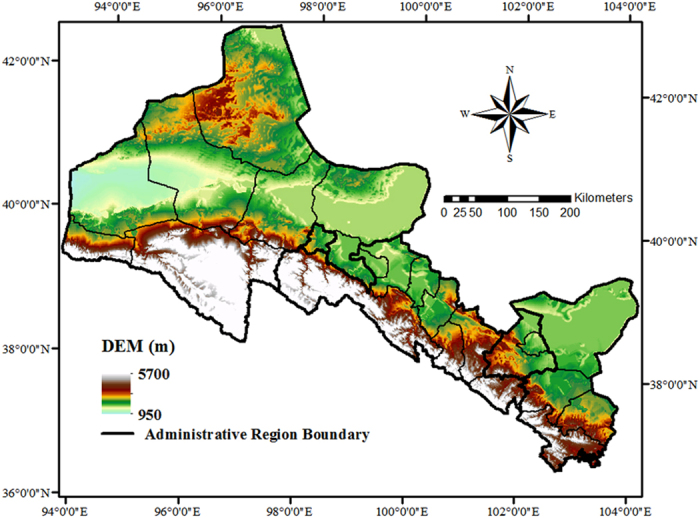
Location of the study area. The map was developed through using ArcGIS 10.0 (http://www.esri.com/software/arcgis/arcgis-for-desktop).

The Hexi Corridor is located in the arid region of Northwest China (92°12′ E-104°20′ E, 37°17′-42°48′ N), with the total area of 270,000 km^2^ and the distance from east to west about 1,000 km (Fig. 1). There are three river systems, Shiyang River, Hei River, and Shule River, from east to west^29^. It is an important agricultural production base of Northwest China, with abundant land, light and heat resources. The climate exhibits typical arid features^30^, with annual mean precipitation of 50–150 mm and annual mean evaporation of 1500–2500 mm. Agricultural production in this region highly relies on irrigation.

### Data collection

Irrigation water productivity of cereal crops is calculated as the yield per unit of irrigation water use (IWP, kg/m^3^). The previous study showed that area supported by unit of irrigation water use (AI, ha/m^3^), amount of fertilization per unit of area (F, kg/ha), amount of agricultural film per unit of area (AF, kg/ha), amount of agricultural pesticide per unit of area (AP, kg/ha) are significantly (*p* < 0.01) correlated with IWP^31^. Therefore, these agronomic practice factors were chosen for further analysis of contributions to the changes of IWP in this study. For the climatic factors, the available precipitation (P, mm), daily mean temperature (T, °C) and solar radiation (R_S_, MJ/m^2^/d), for the crop growing period were considered, as they have been proven to contribute to crop yield and thus IWP^15^,^16^,^17^,^18^.

Irrigation water use is the total irrigation amount applied to cereal crops per unit area (m^3^/ha), and area supported by unit of irrigation water use is the reciprocal of irrigation water use. Fertilization, agricultural film and agricultural pesticide per unit area are calculated as the ratio of the total amount to the crop planting area. And the climatic data are the sum (P) or daily averages (T, R_S_) of the crop growing period, which spans from March 1st to September 30th according to the local situation of the study area.

The statistical data, including the yield of cereal crops, irrigation water use and agronomic practice factors, were collected from the China Economic and Social Development Statistics Database (http://tongji.cnki.net/kns55/index.aspx), Gansu Water Statistical Yearbook, Gansu Development Yearbook and Gansu Rural Yearbook. Daily meteorological data were obtained from China Meteorological Data Network (http://data.cma.cn). The data were available for the period of 1981–2012.

### Statistical analysis

Kendall trend test method was used to detect the changes of IWP and its major driving factors, which has been widely applied to evaluate changing trends of time series in hydrometeorology^32^,^33^. The specific calculation procedure given by Kendall and Syuart^34^ and Mann^35^ is as follows:

For a data series *x*_1_, *x*_2_, …, *x*_*n*_, the number of all pairs of observations that *x*_*i*_ < *x*_*j*_ (*j* > *i*), say *p*, should be determined first. The ordered (*i, j*) subsets are (*i* = 1, *j* = 2, 3, …, *n*), (*i* = 2, *j* = 3, 4, …, *n*) … (*i* = *n* − 1, *j* = *n*), and *n* is the length of data series.

The basic statistic τ is expressed as:


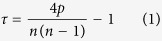


where *E(τ*) = 0; 
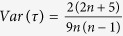
.

The test statistic *N* is defined as


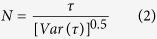


where *N* converges rapidly to a standard normal distribution. And *N*_*1-α/2*_ is a threshold at a given significance level (*α*) that can be obtained from the Standard Normal Distribution Table. If 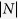 is greater than *N*_1-*α/2*_, the changing trend is statistically significant at the significance level *α*, a positive *N* denotes an increasing trend while negative denotes decreasing.

The concept of average annual growth rate was used to analyze the annual growth range of IWP and its major driving factors. The average annual growth rate is calculated as:


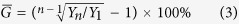


where, 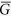 is the average annual growth rate (%), *Y*_*1*_, *Y*_*n*_ is the values of the first and last year of the study period or sub-period, respectively. The correlation analysis was conducted by SPSS 21 software (IBM SPSS Inc., USA).

### Calculation of contribution rate

The contribution rates of driving factors on IWP were calculated by the Cobb-Douglas (CD) production model based on the partial least squares method (PLS). CD production function is proposed by Charles Cobb and Paul Douglas in 1928, which is widely used in practice^36^. The fundamental form of CD production function is expressed as:


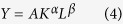


where *Y* is the output, *A* is coefficient, *K* is capital input, *L* is labor input, and *α, β* is the elasticity coefficient of *K* and *L* respectively.

Considering that the driving factors of IWP are more than two, the following CD production model was developed:


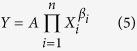


where *X*_*i*_is the *i*th factor and *β*_*i*_ is the elasticity coefficient of *X*_*i*_.

Linearizing the model by taking the logarithm for parameter estimation:


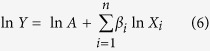


The function after linearization (Eq. 6) is the standard multivariate linear function which can be easily solved by multiple linear regression method based on the historical data. In this study, partial least squares method (PLS) was used for estimating parameters in order to avoid multicollinearity of the driving factors.

Through differential computation of Eq. 6, the contribution rate of the *i*th factor, i.e. *E*_*i*_ is expressed as:


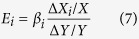


Assessment criteria, namely mean absolute error (MAE), mean relative error (MRE) and root mean square error (RMSE), were adopted to evaluate the performance of the model. The three criteria are expressed as:


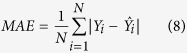



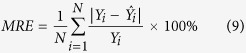



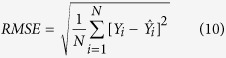


where *N* represents the number of sampling points, *Y*_*i*_, 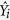 represents the original and predicted values respectively.

## Results and Discussion

### Trends of IWP and the driving factors

**Figure 2:**
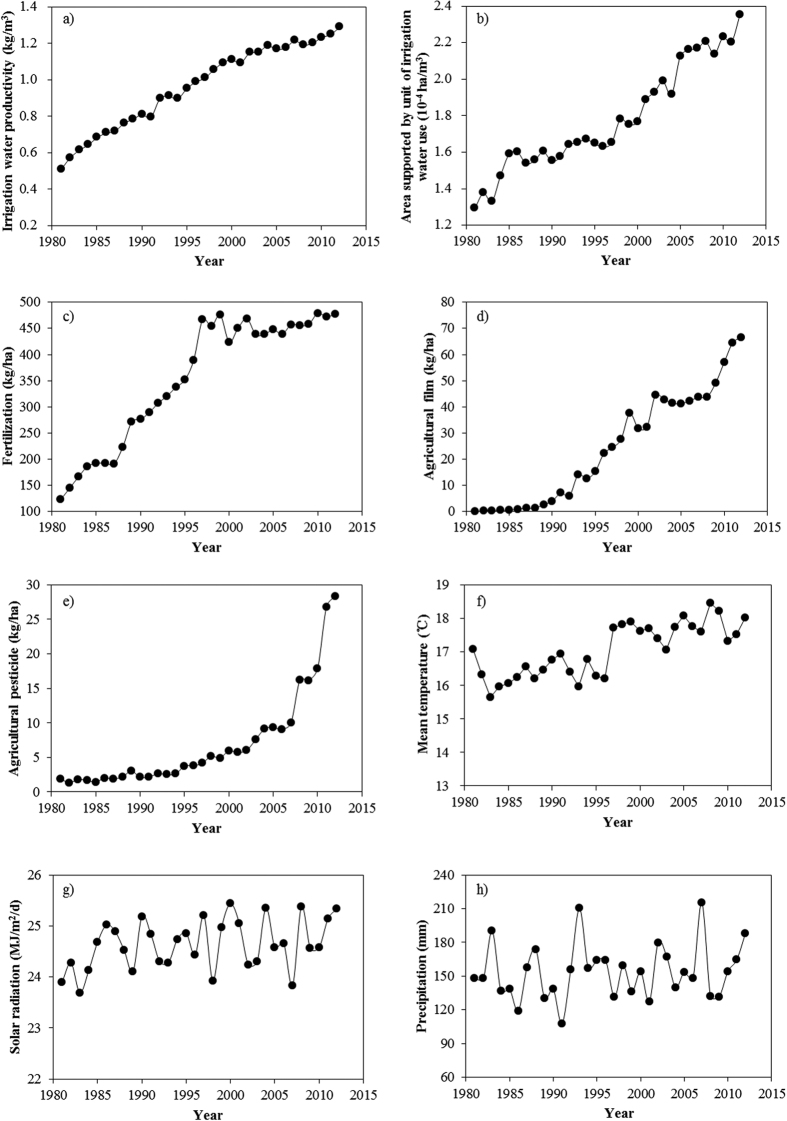
Changes of irrigation water productivity and its driving factors for the period of 1981–2012.

The time series of IWP and its driving factors from 1981 to 2012 averaged over the whole region are plotted in Fig. 2. As shown in Fig. 2, IWP and its driving factors increased overall for the past 32 years, while they show different patterns during the study period. IWP, area supported by unit of irrigation water use (AI) and amount of fertilization per unit of area (F) obviously increased all along the time (Fig. 2a–c). The amount of agricultural film per unit of area (AF) and amount of agricultural pesticide per unit of area (AP) both had a slight increase in the early years while grew rapidly in the late period (Fig. 2d,e). All these agronomic practice factors significantly increased (*p* < 0.01) (Table 1 column 1 and column 2), while daily mean temperature (T) and solar radiation (R_S_) presented slow increasing trends with notable fluctuations (Fig. 2f,g), and precipitation (P) increased slightly and non-significantly (Fig. 2h, Table 1 column 1 and column 2). The results were similar with the study of Meng *et al*.^37^, in which the annual mean temperature displayed a statistically significant increase with a rate of 0.27 °C/10a and the increasing trend of annual precipitation was not significant during the period 1955–2011.

Average annual growth rate was calculated for both the sub-periods and the whole period (1981–2012), for further understanding the changes of the indicators (Table 1). From 1981 to 1989 (Period 1), IWP increased by 5.59% of that in 1981, and increased by 3.41%, 1.24% for period of 1990–1999 (Period 2) and 2000–2012 (Period 3), respectively. The average annual growth rate of AI was 2.74%, 1.36% and 2.42% for Period 1, Period 2 and Period 3, respectively, and for F, the rate was 10.41%, 6.23% and 0.99% respectively, which slowed down greatly for the recent years. In terms of AF, the average annual growth rate reached to 67.27%, 28.46% and 6.34%, indicating a large increasing percentage in the early period but a slow increase in the late period. The average annual growth rate of AP was 5.88%, 9.77% and 13.86% respectively. Changes of T, R_S_ and P showed fluctuations, with the maximum increase rates appearing in Period 2, Period 1 and Period 3, respectively and largest decrease rates in Period 1, Period 2 and Period 1 respectively. On the whole, they showed much smaller increase rates compared to the agronomic practice factors.

### Correlation between IWP and major driving factors

**Figure 3:**
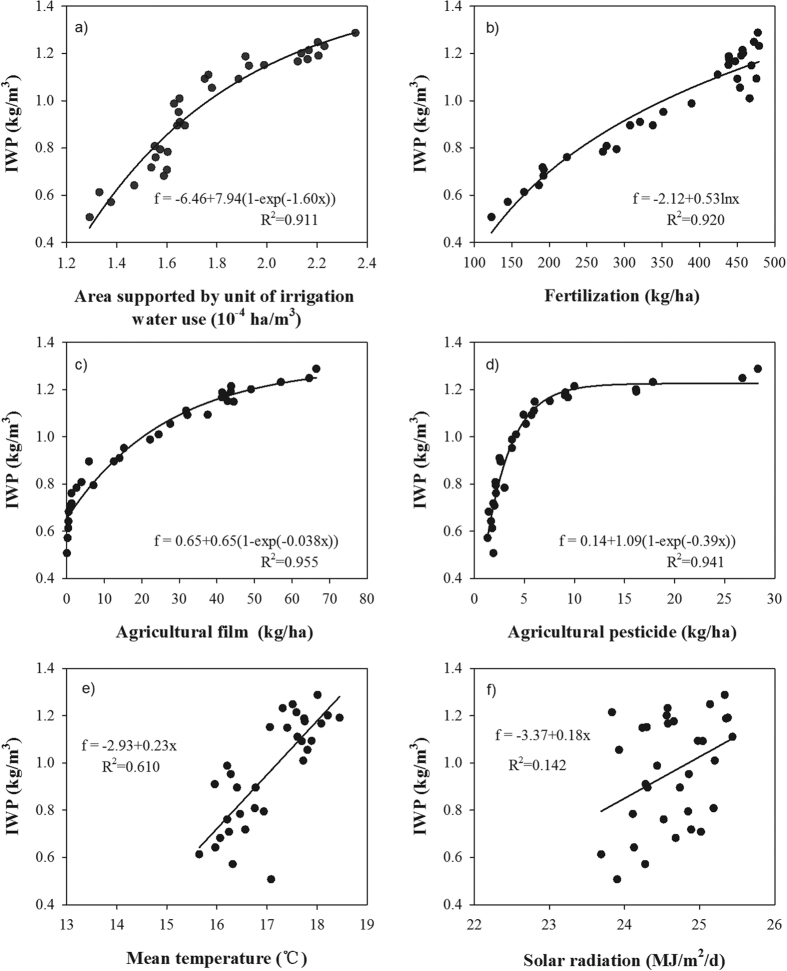
Relationships between irrigation water productivity (IWP) and its significantly correlated driving factors.

The results of correlation analysis(*R*, adjusted *R*^*2*^, and significance) are provided in Table 2. AI, F, AF, AP and T showed relatively greater correlations with IWP, while R_S_ came next and P was poorly related to IWP. In general, all the agronomic practice factors and T showed statistically significant correlations with IWP at the level of 0.01 and R_S_ significantly correlated with IWP at the level of 0.05, while P has no significant correlation with IWP. Figure 3 shows the relationships between IWP and each of its significantly correlated driving factors, e.g. AI, F, AF, AP, T and R_S_. The curves of IWP versus each factor demonstrated IWP increased with the increasing of each factor, but the curves were in various shapes and for the same agronomic practice factor, the trend was different in different ranges.

The correlation patterns between IWP and AI and that between IWP and F were similar. IWP increased with the increasing of AI and F, while the increase slowed down slightly when AI and F became larger (Fig. 3a,b). IWP increased with both AF and AP, but the changes were different along the time. IWP increased rapidly when the AF and AP were small, and IWP grew slowly as AF and AP were larger (Fig. 3c,d); moreover, the increasing rate was gradually steady and the value of IWP changed little when AF and AP reached to a certain extent. It indicated that the input amounts of AF and AP were relatively oversupplied and for improving IWP the appropriate application of AF and AP should be considered. Positive linear correlations were also found between T and IWP and also between R_S_ and IWP (Fig. 3e,f).

The results of correlation analysis were consistent with previous researches. In the arid region, irrigation is one of the main factors that influence crop growth^38^; therefore, it showed strong correlation with IWP. Ali and Talukder^12^ showed that IWP increased with the decreasing of irrigation water; AI, the inverse of irrigation water use per unit area, certainly positively correlated with IWP. Agricultural film, as one of ways for soil surface modification, has a great effect on promoting plant growth and increasing yield by changing the processes of the energy and water balance components of crop growth system^19^,^39^. Agricultural film has been widely used in northern China for many years and it contributes to increase topsoil temperature for early growth, maintain soil water content, and promote grain yield and water productivity^40^,^41^. Nutrient limitation affects yield potential obviously, and fertilization will improve soil nutrient and the improved soil nutrient status certainly increases water productivity^42^. The improvement of crop growth and yield benefited by proper fertilization could result in the increases in IWP^5^,^19^. Hu *et al*.^25^ found the positive correlation between IWP and temperature in Zhangye Region of Hexi Corridor, as is the same with ours. Solar radiation promotes crop growth and development and has a linear relationship with the dry matter production^15^.

### Contribution rate

**Figure 4:**
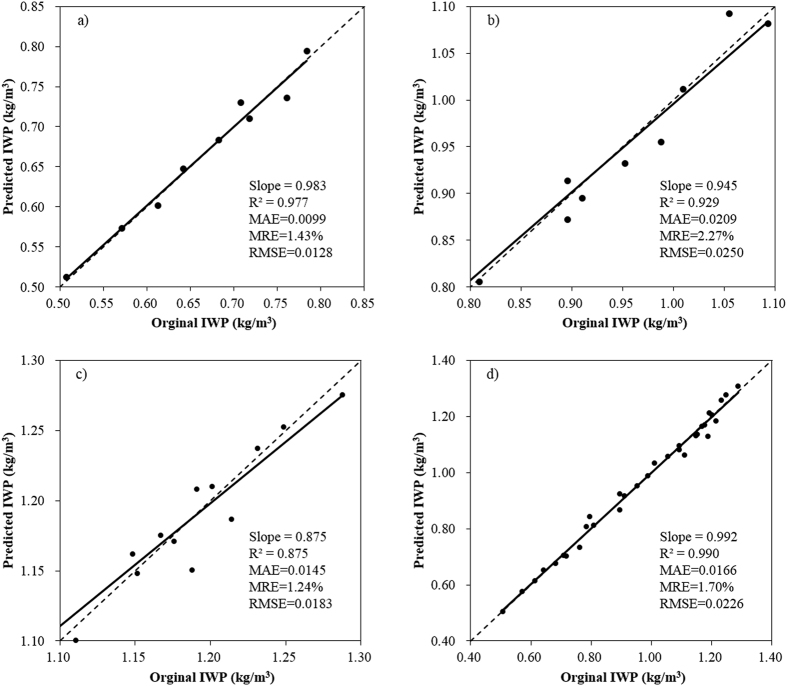
Comparison between the predicted and original values of IWP for each period (**a**, 1981–1989; **b**, 1990–1999; **c**, 2000–2012; **d**, 1981–2012), MAE is mean absolute error (kg/m^3^), MRE is mean relative error (%), and RMSE is root mean square error (kg/m^3^).

CD production function models based on PLS method (PLS-CD models) were developed for the whole period (1981–2012) and three sub-periods, i.e., 1981–1989, 1990–1999 and 2000–2012, in order to analyze the generalized relationship between IWP and the driving factors. The model performances were quantified using coefficient of determination (*R*^*2*^), which was 0.982, 0.925, 0.870 and 0.992 respectively for the period of 1981–1989, 1990–1999, 2000–2012 and the whole period, indicating good estimations. Comparisons between the predicted and original IWP values are shown in Fig. 4, in which the assessment criteria are provided. The predicted IWP well matched the original data, and the assessment indicators showed good performances of the developed models in all periods. Therefore, PLS-CD models developed in different periods have strong ability in reflecting the relationships between IWP and the driving factors; the changes of IWP can be well explained by the changes of the driving factors.

**Figure 5:**
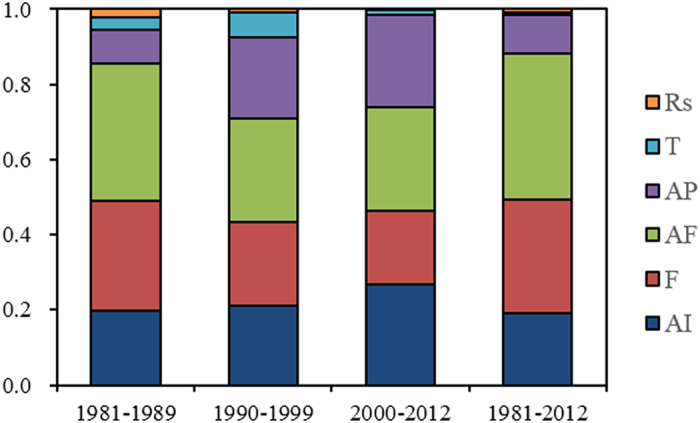
Proportions of the contribution rates of each driving factor in different periods, AI: area supported by unit of irrigation water use, F: fertilization per unit of area, AF: agricultural film per unit of area, AP: agricultural pesticide per unit of area, T: daily mean temperature for crop growing period, R_S_: daily solar radiation for the crop growing period.

Table 3 shows the contribution rates of the driving factors on IWP in different periods, based on the developed PLS-CD models. The contribution rate here refers to the effect of the changes of driving factors on the increase of IWP. Proportions of the contribution rates for driving factors are also presented in Fig. 5 and the sum of total rates for one period is set to 1. On the whole, the contribution of the controllable agronomic practice factors is greater than that of the climatic factors. For the whole period, the agronomic practice factors including AI, F, AF and AP contributed 20.6%, 32.8%, 42.3% and 11.1% respectively to the increase of IWP; and the contribution rates of the climatic factors, i.e. T and R_S_, are −0.9% and 0.9%. T contributed slightly negatively to the increase of IWP during the whole period, resulting from the negative elasticity coefficient of T to IWP and positive growth of both T and IWP in this period. The contribution rates of the factors changed in different sub-periods. AF had the largest contribution rate of 40.1% in Period 1; reduced to 27.8% in Period 2, and a slightly increase was found in Period 3. The contribution rate of F presented a decreasing trend, with 32.0% for Period 1, 22.5% for Period 2, and 20.0% for Period 3. AI showed obvious growth from 21.4% of Period 2 to 27.2% of Period 3. The contribution rate of AP in Period 1 was small (10.0%); and had a large increase to 21.7% in Period 2 and reached to the highest in Period 3 (24.8%). At the early stage, the amount of agricultural film and fertilization per unit area increased a lot from being rarely used, which obviously promoted the yield. Thus the contribution rate of AF and F were larger in the Period 1. With the increase of yield and IWP slowing down, the contribution rates of AF and F gradually stabilized in the later years, when the use of agricultural film was close to saturation and fertilization was even in an excess. However, AP increased a little in Period 1 (Table 1); in consequence, the contribution rate of AP was smaller in this period. It can be concluded that the agronomic practices i.e. AI, F, AF and AP are the dominant factors that contribute to the changes of IWP for all the sub-periods (Table 3). The contribution rates of T and R_S_ in each sub-period were quite small compared with the other factors. For the period of 2000–2012, R_S_ showed a negative contribution rate and its absolute value was quite small (−0.04%), which indicates that during this period R_S_ slightly affected the growth of IWP negatively.

It should be noted that after the year of 2000 the growth rate of IWP slowed down (Table 1), although F, AF and AP contributed great proportions (Fig. 5) and increments of their inputs were larger than earlier years (Fig. 2). Figure 3 illustrates that the increasing of IWP was not obvious and gradually became steady when the agronomic inputs, e.g. fertilization, agricultural film and agricultural pesticide, reached to a certain extent. It has been demonstrated that the yield does not remarkably increase with the increasing input of fertilization per unit of area, due to the excessive application of fertilizer and lack of scientific and technical guidance^43^. Thus, from the perspective of agricultural production, it is critical that the increase of agronomic inputs should be carefully considered and the optimum amount of the inputs should be applied based on a comprehensive assessment in order to improve IWP. In the Hexi Corridor, improvement of IWP should rely on advanced water-saving technology and application of optimum (need-based) fertilizer, agricultural film and pesticide, ensuring efficient use of agronomic inputs instead of simply increasing the input amount.

## Conclusions

IWP and most of the driving factors significantly increased along the time except for precipitation, with different average annual growth rates in each sub-period. And there were statistically significant correlations between IWP and the driving factors except for precipitation. The PLS-CD models developed in different periods had good performances. The results of contribution rates indicated that irrigation water productivity in the Hexi Corridor is more sensitive to agronomic practice factors than climatic factors. The agronomic practice factors are predominant factors on the changes of IWP: AF had largest contribution in the first stage and tended to be stable with time; AI and F had great influence all the time; and the contribution rate of AP was small in the early stage but increased in the subsequent sub-periods. The impacts of mean temperature and solar radiation, which are the uncontrollable natural factors, were weak during the whole period.

Moreover, it was found that the continued increasing amount of agronomic inputs no longer has apparent effect on the improvement of IWP. Thus, in the Hexi Corridor motivation policies for maximizing IWP should be considered carefully. IWP and food production should be improved through promoting water-saving irrigation technology, maintaining the current use of fertilization, agricultural film and agricultural pesticide and improving the use efficiencies of agronomic inputs, instead of increasing the amount. This study provides the basis for exploring ways of improving irrigation water productivity, and more specific schemes still need to be developed.

## Additional Information

**How to cite this article**: Li, X. *et al*. Irrigation water productivity is more influenced by agronomic practice factors than by climatic factors in Hexi Corridor, Northwest China. *Sci. Rep.* **6**, 37971; doi: 10.1038/srep37971 (2016).

**Publisher's note:** Springer Nature remains neutral with regard to jurisdictional claims in published maps and institutional affiliations.

## Figures and Tables

**Table 1:** Kendall test and average annual growth rates of irrigation water productivity and its driving factors in different periods.

Indicator	*N*-value	Trend	Average annual growth rate(%)			
			1981–1989	1990–1999	2000–2012	1981–2012
Irrigation water productivity (IWP)	7.751	↑^**^	5.59	3.41	1.24	3.05
Area supported by unit of irrigation water use (AI)	7.103	↑^**^	2.74	1.36	2.42	1.96
Fertilization per unit of area (F)	6.616	↑^**^	10.41	6.23	0.99	4.48
Agricultural film per unit of area (AF)	7.524	↑^**^	67.27	28.46	6.34	26.77
Agricultural pesticide per unit of area (AP)	7.297	↑^**^	5.88	9.77	13.86	9.08
Daily mean temperature for the crop growing period (T)	4.378	↑^**^	−0.46	0.73	0.19	0.17
Daily solar radiation for the crop growing period (R_s_)	2.076	↑^*^	0.11	−0.09	−0.03	0.19
Precipitation for the crop growing period (P)	0.941	↑	−1.57	−0.16	1.67	0.78

*N*-values are values of test statistic from Kendall trend test based on the regional averaged agronomic practice and climatic data; ↑^*^ and ↑^**^indicate an increasing trend at the significance levels of 0.05 and 0.01, respectively.

**Table 2:** The univariate correlation between irrigation water productivity and its driving factors.

Factor	*R*	Adjusted *R*^2^	Significance
Area supported by unit of irrigation water use (AI)	0.926	0.853	ES
Fertilization per unit of area (F)	0.971	0.940	ES
Agricultural film per unit of area (AF)	0.946	0.891	ES
Agricultural pesticide per unit of area (AP)	0.742	0.535	ES
Daily mean temperature for the crop growing period (T)	0.782	0.598	ES
Daily solar radiation for the crop growing period (R_S_)	0.378	0.114	S
Precipitation for the crop growing period (P)	0.192	0.005	NS

*R*: correlation coefficient, Adjusted *R*^2^: adjusted coefficient of determination.

ES: extremely significant at the 0.01 level, S: signiciant at the 0.05 level, NS: not signiciant.

**Table 3:** Results of PLS-CD production function models and the contribution rate of each driving factor to the changes of IWP.

Factor	1981–1989		1990–1999		2000–2012		1981–2012	
	*β*	E (%)	*β*	E (%)	*β*	E (%)	*β*	E (%)
Area supported by unit of irrigation water use (AI)	0.444	21.8	0.538	21.4	0.139	27.2	0.322	20.6
Fertilization per unit of area (F)	0.172	32.0	0.123	22.5	0.249	20.0	0.223	32.8
Agricultural film per unit of area (AF)	0.033	40.1	0.033	27.8	0.055	28.4	0.048	42.3
Agricultural pesticide per unit of area (AP)	0.095	10.0	0.076	21.7	0.022	24.8	0.037	11.1
Daily mean temperature for the crop growing period (T)	−0.439	3.6	0.315	6.7	0.109	1.6	−0.152	−0.9
Daily solar radiation for the crop growing period (R_S_)	1.185	2.3	−0.349	1.0	0.014	−0.04	0.147	0.9

*β* is the parameter estimated by PLS-CD production model, i.e. the elasticity coefficient, E(%) is the contribution rate.
